# Comparative analysis of the complete chloroplast genome among *Prunus mume*, *P*. *armeniaca*, and *P*. *salicina*

**DOI:** 10.1038/s41438-019-0171-1

**Published:** 2019-07-21

**Authors:** Song Xue, Ting Shi, Wenjie Luo, Xiaopeng Ni, Shahid Iqbal, Zhaojun Ni, Xiao Huang, Dan Yao, Zhijun Shen, Zhihong Gao

**Affiliations:** 1 College of Horticulture, Nanjing Agricultural University, 210095 Nanjing, China; 2 Jiangsu Key Laboratory for Horticultural Crop Genetic Improvement, Nanjing, China

**Keywords:** Evolution, Genome

## Abstract

*Prunus mume* Sieb. et Zucc., *P*. *armeniaca* L., and *P*. *salicina* L. are economically important fruit trees in temperate regions. These species are taxonomically perplexing because of shared interspecific morphological traits and variation, which are mainly attributed to hybridization. The chloroplast is cytoplasmically inherited and often used for evolutionary studies. We sequenced the complete chloroplast genomes of *P. mume, P. armeniaca*, and *P. salicina* using Illumina sequencing followed by de novo assembly. The three chloroplast genomes exhibit a typical quadripartite structure with conserved genome arrangement, structure, and moderate divergence. The lengths of the genomes are 157,815, 157,797, and 157,916 bp, respectively. The length of the large single-copy region (LSC) region is 86,113, 86,283, and 86,122 bp, and the length of the SSC region is 18,916, 18,734, and 19,028 bp; the IR region is 26,393, 26,390, and 26,383 bp, respectively. Each of the three chloroplast genomes encodes 133 genes, including 94 protein-coding, 31 tRNA, and eight rRNA genes. Differential gene analysis for the three species revealed that *trnY-ATA* is a unique gene in *P. armeniaca*; in contrast, the gene *trnI-TAT* is only present in *P. mume* and *P. salicina*, though the position of the gene in these chloroplast genomes differs. Further comparative analysis of the complete chloroplast genome sequences revealed that the ORF genes and the sequences of linked regions *rps16* and *atpA*, *atpH* and *atpI*, *trnc-GCA* and *psbD*, *ycf3* and *atpB*, and *rpL32* and *ndhD* are significantly different and may be used as molecular markers in taxonomic studies. Phylogenetic evolution analysis of the three species suggests that *P. mume* has a closer genetic relationship to *P. armeniaca* than to *P. salicina*.

## Introduction

The evolutionary process occurring in stone fruit trees is an interesting topic. However, phylogenetic relationships among *P. mume*, *P. armeniaca*, and *P. salicina* have been problematic because of frequent hybridization, apomixis, presumed rapid radiation, and complex historical diversity. Genome sequencing is frequently used to analyze phylogenetic relationships, genetic diversity, and evolutionary studies^1^. Three independent genomes offering genetic information are those of the chloroplast, mitochondrion, and nucleus. Compared with the nuclear genome, the chloroplast genome has a small size, single-parental inheritance, low nucleotide substitution rate, haploid nature, and highly conserved genomic structure^2,3^. Therefore, the chloroplast genome has been considered the perfect model for diversity and evolution studies.

The development of the chloroplast in plants is proposed to have initiated from multiple endosymbiosis of cyanobacteria and photosynthesis vectors^4^. The chloroplast is an organelle that exists in the cytoplasmic matrix and is enveloped by a bilayer membrane, with a flat ellipsoidal or spherical shape. In addition to photosynthesis, chloroplasts are involved in the synthesis of starch, fatty acids, pigments, and amino acids. Chloroplasts are also semi-autonomous genetic organelles and contain independent chloroplast DNA (cpDNA). The first chloroplast genome sequencing of tobacco was completed in 1986^5^, and at the end of March 2018, there were 13,602 complete plant chloroplast genomes collected at the National Center for Biotechnology Information (NCBI). In general, chloroplast DNA has a double-stranded, circular, typically four-segment structure, with a few linear molecular structures. It contains a LSC, a small single-copy region (SSC), and a pair of reverse complementary repeat regions (IRs), with the IR region separating the LSC and SSC regions. The length of the genome is ~120–160 kb^6^, and differences are mostly due to IR expansion/contraction or loss^7,8^. For example, the chloroplast genome of some algae does not contain an IR region^9,10^. Some leguminous plants lose one of the IR regions^10^, whereas the chloroplast genome of *Pisum sativum* has lost the IR segment, which shortened its length. The chloroplast genome generally encodes 110–130 genes, which are highly conserved with regard to composition and sequence. Furthermore, the conserved structure of cpDNA and its low nucleotide substitution rate play a vital role in phylogenetic studies^11^.

The Rosaceae family contains over 120 genera and 3300 species with a great economic importance widely distributed in temperate regions^12^. The family can be divided into four subfamilies according to fruit type: Rosoideae, Prunoideae, Spiraeoideae, and Maloideae, with *P. mume*, *P. armeniaca*, and *P. salicina* belonging to Prunoideae. Nuclear genome sequences have been published for *Rosa* and *Malus* *×* *domestica*^13^, seven species of the genus *Firago*^14^, and *Rubus occidentalis*^15^, providing valuable information for evolutionary classification. However, due to apomixis, hybridization, and hypothetical rapid radiation, the phylogenetic relationship among Rosaceae species is complex^12^. The chloroplast genome of *P. mume*, *P. armeniaca*, and *P. salicina* can be used to understand the structure and rapid evolution of the *Prunus* genome, which will also help to illustrate and assess chloroplast genetic diversity. A profound analysis of phylogenetic relationships in *Prunus* species through chloroplast genome sequences would be valuable and interesting.

In this study, we completely sequences the chloroplast genomes of *P. mume*, *P. armeniaca*, and *P. salicina* using Illumina technology followed by reference-guided assembly of de novo contigs. Furthermore, we compared the chloroplast genomes of these three species with the complete chloroplast sequence of 23 other Rosaceae species and constructed a phylogenetic tree, which was further used for exploring genetic relationships among *P. mume*, *P. armeniaca*, and *P. salicina* using the entire chloroplast genome, coding regions, LSCs, IRs, and introns.

## Results

### Characterization of chloroplast genomes in *Prunus* species

**Fig. 1:**
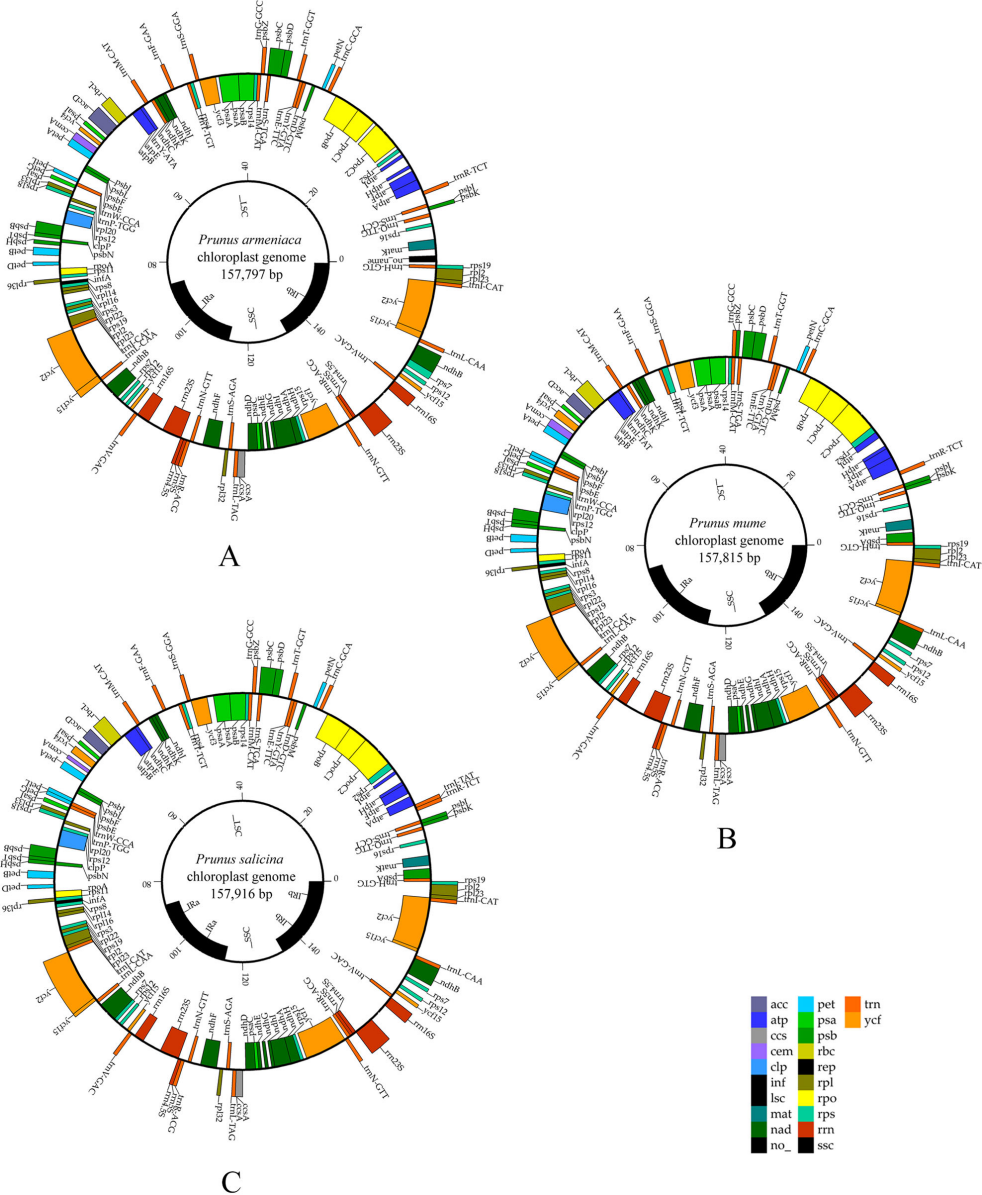
Chloroplast genome maps of three *Prunus* species. **a** *P. salicina* chloroplast genome. **b** *P. mume* chloroplast genome. **c** *P. armeniaca* chloroplast genome. Genes shown outside the circle are transcribed clockwise and those inside counterclockwise. Genes belonging to different functional groups are color-coded

A total of 27.49 Gb clean data were obtained after sequencing; regarding statistical assessment of base quality, we obtained 94.17% of Q30 bases. For *P. mume*, *P. armeniaca*, and *P. salicina*, 1,107,094, 662,524, and 824,216 paired-end reads and 297, 296, and 298 bp, respectively, of average insert size were produced by Illumina sequencing. The average organelle coverage for *P. mume*, *P. armeniaca*, and *P. salicina* with the reference genome reached 1059, 634, and 788, respectively. The chloroplast genomes of the three *Prunus* species along with their size, reads, GC contents, and average are shown in Table 1. The entire genome size of *Prunus* species is similar to the reference peach genome, at ~160 kb. We obtained complete chloroplast genome maps of *P. mume*, *P. armeniaca*, and *P. salicina* through de novo genome sequencing and assembly (Fig. 1).

**Table 1:** Summary statistics for the assembly of five *Prunus* species chloroplast genomes

Genome features	*P. armeniaca*	*P. mume*	*P. salicina*	*P. pseudocerasus*	*P. persica*
Genome size (bp)	157,797	157,815	157,916	157,834	157,790
LSC size (bp)	86,283	86,113	86,122	85,964	85,968
SSC size (bp)	18,734	18,916	19,028	19,084	19,060
IR size (bp)	26,390	26,393	26,383	26,393	26,381
Number of genes	133 (110)	133 (110)	133 (110)	131 (111)	130 (110)
Protein genes [unique]	94 (80)	94 (80)	94 (80)	86 (78)	85 (77)
tRNA genes [unique]	31 (26)	31 (26)	31 (26)	37 (29)	37 (29)
rRNA genes [unique]	8 (4)	8 (4)	8 (4)	8 (4)	8 (4)
Duplicated genes in IR	18	18	18	16	14
GC content (%)	36.75	36.74	36.74	37	37
GC content in LSC (%)	34.54	34.58	34.58	35	35
GC content in SSC (%)	30.43	30.35	30.40	30	30
GC content in IR (%)	42.57	42.56	42.59	43	43
Total reads	23,517,590	44,218,598	23,901,827	–	–
Aligned paired-end reads	662,524	1,107,094	824,216	–	–
Assembled reads	362,494	468,122	419,364	–	–
Average organelle coverage	634	1059	788	–	–
Average insert size (bp)	296	297	298	–	–

**Fig. 2:**
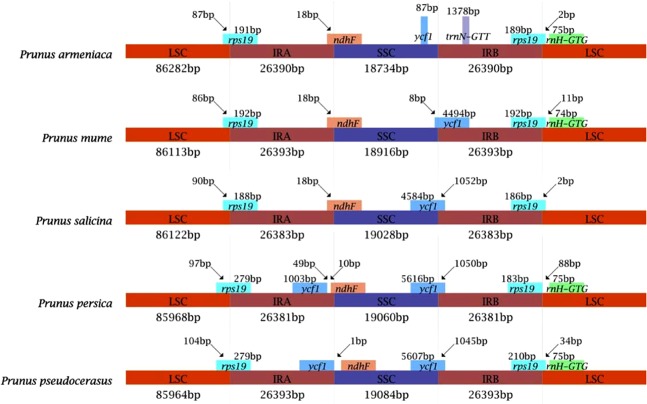
Comparison of the borders of LSC, SSC, and IR regions of chloroplast genomes in five *Prunus* species

The chloroplast genomes of *P. armeniaca*, *P. mume*, and *P. salicina* exhibit a typical quadripartite structure with conserved genome arrangement, structure, and divergence and are similar to those of *P. persica* and *P. pseudocerasus*. The chloroplast genome size is ~157 kb in *Prunus* species, including a pair of IRs separated by an LSC region and an SSC region (Table 1 and Fig. 2). The GC content of these *Prunus* chloroplast genomes is ~37% (Table 1); the GC content of the IR regions is ~43% and those of LSC and SSC regions ~35% and 30%, respectively. These results lead us to infer that the LSC, SSC, and IR regions of five *Prunus* species are similar but that the GC contents are higher in IR regions due to the high GC contents of eight rRNA genes distributed in these regions.

The chloroplast genes of *Prunus* species contain 133 genes (110 unique genes), including 94 protein-coding, 31 tRNA, and eight rRNA genes (Table 1 and Table 2). There are 18 duplicated genes, including four rRNA genes and 13 other genes (*ycf2*, *ycf15*, *rpl2*, *rps19*, *trnI-CAT*, *rpl23*, *trnL-CAA*, *ndhB*, *rps7*, *rps12*, *trnV-GAC*, *trnR-ACG*, and *trnN-GTT*) repeats once where the ycf15 gene repeats twice in the IR region. Furthermore, 12 intron-containing genes were found (Table 3), including nine different genes (*psaA*, *atpF*, *rpl22*, *ndhA*, *ndhB*, *rpoC1*, *trnS*-*AGA*, *rpl2*, and *ycf15*) containing one intron and *trnI-TAT* in *P. salicina* and *P. mume*. The *trnI-ATA* gene in *P. armeniaca* also contains one intron. Two genes (*ycf3* and *clpP*) have two introns. These nine introns are located in the LSC region; two introns are in the SSC region and three introns in the IR region. The complete chloroplast genome with gene annotations has been submitted to NCBI under GenBank accession numbers MH700953 for *P. mume*, MH700954 for *P. armeniaca*, and MH700952 for *P. salicina*.

**Table 2:** List of annotated genes in *P. mume*, *P. armeniaca*, and *P. salicina* chloroplast genomes

Category	Group of gene	Name of gene
Photosynthetic	Subunits of photosystem I	*psaA(x2), psaB, psaC, psaI, psaJ*
	Submits of photosystem II	*psbA, psbB, psbC, psbD, psbE, psbF, psbH, psbI, psbJ, psbK, psbL, psbM, psbN, psbT, psbZ*
	Subunits of NADH dehydrogenase	*ndhA, ndhB(x2), ndhC, ndhD, ndhE, ndhF, ndhG, ndhH, ndhI, ndhJ, ndhK(x2)*
	Subunits of cytochrome b/f complex	*petA, petB, petD, petG, petL, petN*
	Subunits of ATP synthase	*atpA, atpB, atpE, atpF, atpH, atpI*
	Large subunit of rubisco	*rbcL*
Self-replication	Proteins of large ribosomal subunit	*rpl2(x2), rpl14, rpl16, rpl20, rpl22, rpl23(x2), rpl32, rpl33, rpl36*
	Proteins of small ribosomal subunit	*rps2, rps3, rps4, rps7(x2), rps8, rps11, rps12(x3), rps14, rps15, rps16, rps18, rps19(x2)*
	Subunits of RNA polymerase	*rpoA, rpoB, rpoC1, rpoC2*
	Ribosomal RNAs	*rrn23S(x2), rrn16S(x2), rrn5S(x2), rrn4.5S(x2)*
	Transfer RNAs	trnR-TCT, trnY-ATA*, trnC-GCA, trnT-GGT, trnI-CAT(x2), trnS-GGA, trnF-GAA, trnM-CAT, trnG-GCC, trnR-ACG(x2), trnL-TAG, trnH-GTG, trnY-GTA, trnP-TGG, trnV-GAC(x2), trnS-GCT, trnS-AGA, trnQ-TTG, trnD-GTC, trnL-CAA(x2), trnW-CCA, trnT-TGT, trnfM-CAT, trnS-TGA, trnN-GTT(x2), trnE-TTC
Biosynthesis	Maturase	*matK*
	Protease	*clpP*
	Envelope membrane protein	*cemA*
	Acetyl-CoA carboxylase	*accD*
	c-type cytochrome synthesis gene	*ccsa(x2)*
	Translation initiation factor	*infA*
Unknown function	Conserved hypothetical chloroplast Reading Frames	*ycf1, ycf2(x2), ycf3, ycf4, Ycf15(x4)*

Asterisk denotes the *trnY-ATA* gene is *trnY-ATA* in *P. armeniaca* but is *trnI-TAT* in *P. mume* and *P. salicina*

**Table 3:** Information on 12 intron-containing genes in the chloroplast genome of *Prunus* species

Gene	Location	Exon I (bp)	Intron I (bp)	Exon II (bp)	Intron II (bp)	Exon III (bp)
*trnI-TAT*	LSC	38	83	42		
*psaA*	LSC	1787	31	323		
*ycf3*	LSC	126	713	229	764	149
*atpF*	LSC	147	683	466		
*rpl2*	IR	384	648	469		
*ycf15*	IR	200	295	110		
*clpP*	LSC	73	805	296	648	222
*rpl22*	LSC	380	64	123		
*ndhA*	SSC	555	1147	535		
*ndhB*	IR	869	588	752		
*rpoC1*	LSC	455	755	1613		
*trnS-AGA*	SSC	48	77	34		

Shrinkage and expansion of the IR region is an important aspect of the chloroplast genome, which is the main reason for the different sizes of these genomes. The IR regions of the five species of *Prunus* are shown in Fig. 2. The gene content and arrangement of the five species are the same in the IR region, which is extended in the *rps19* and *ycf1* genes. The *rps19* gene, located at the boundary of the LSC/IRa region of the five *Prunus* species, shows the same fragment size of 278 bp in all species. In the LSC region, the fragment size ranges from 87 to 90 bp; in the SSC region, the fragment size ranges from 188 to 191 bp. The difference in the boundary region is one of the main reason for differences in chloroplast genome sizes. In addition, the IRa/SSC boundary is crossed by the *ndhF* gene, with equal distributions in *P. mume* and *P. armeniaca* of 18 bp in IRa, and 2219 bp in SSC. The *ndhF* gene in the *P. salicina* chloroplast genome spans the boundary of the IRa/SSC region, with 3 bp more than in *P. armeniaca* and *P. mume*. Based on IRa/LSC and IRa/SSC boundaries, the relationship between *P. mume* and *P. armeniaca* is closer than that between *P. mume* and *P. salicina*. At the SSC/IRb border, *ycf1* is a critical gene that spans the IRb region and the SSC region in *P. salicina* and *P. mume*. The sizes of the fragments in the SSC regions of *P. mume* and *P. salicina* are 8 bp and 4584 bp, respectively. The size of the *ycf1* gene fragment located in the IRb region is 4494 bp in *P. mume* and 1052 bp in *P. salicina*. The *ycf1* gene located at the SSC region is only 87 bp from the SSC/IRb boundary in *P. armeniaca*. Furthermore, the *trnN-GTT* gene located in the IRb region is 1378 bp from the critical point. At the IRb/LSC boundary, the *rps19* gene spans two regions in the three species with similar fragment sizes between the two regions. The fragment sizes of *P. mume*, *P. salicina*, and *P. armeniaca* are 192, 186, and 189 bp; those of the LSC region are 11, 2, and 2 bp, respectively. By comparing the IRb/SSC and LSC/IRb regions of *rps19*, *ndhF*, and *ycf1*, significant differences in fragment lengths of SSC and IRb regions were found among the three species.

### Repeat sequence and codon analysis

**Fig. 3:**
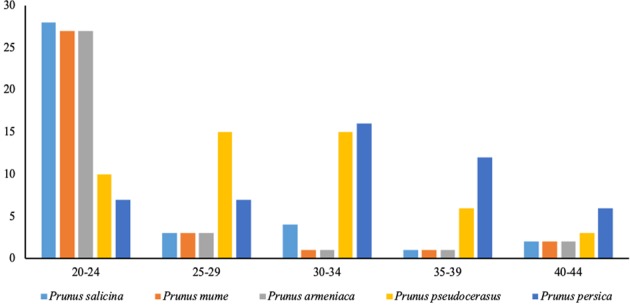
Length distribution of repeat sequences in *Prunus* species

REPuter software was used to identify a large number of repeat sequences in the chloroplast genome of *Prunus* species (Table 4). These repeats are distributed from 20 to 40 bp in the gene spacer (*psbT to psbN*, *trnT* to *TGT-trnF-GAA*, *psbI* to *trnS-GCT*, and *rps19* to *trnH-GTG*), the coding region (*rps12*, *ndhK*, *trnS*-*TGA*, and *ndhC*), introns (*ndhA*, *trnS-AGA*, *trnS-AGA*, and *Ycf3*) and other regions. In particular, the *ycf3* intron region and the *rps19*-*trnH*-*GTG* spacer region exhibit multiple nested sequence repeats. The chloroplast genomes of *P. mume*, *P. armeniaca*, and *P. salicina* have 10, 14, and 12 forward repeats, 13, 13, and 15 palindrome repeats, and 11, 12, and 11 reverse repeats, respectively, with no complementary repeats. The length distribution of the repeat sequence is mainly 20–24 bp and rarely 35–39 bp among *P. mume, P. salicina*, and *P. armeniaca* (Fig. 3). However, a significant difference among these five accessions, *P. persica* and *P. pseudocerasus* was found, whereby the difference in the number of repeats among the three species is greatest at 20–24 bp repeats, whereas *P. persica* and *P. pseudocerasus* have the highest number of repeats at 30–34 bp.

**Table 4:** Summary of repeat sequences and SSRs in five *Prunus* accessions

Species	*P. armeniaca*	*P. mume*	*P. salicina*	*P. persica*	*P. pseudocerasus*
Total number	39	34	38	48	49
Forward	14	10	12	18	19
Palindromic	13	13	15	20	20
Reverse	12	11	11	10	10
SSR loci (N)	59	54	49	57	57
P1^a^ locia (N)	52	49	44	51	48
P2^b^ loci (N)	4	4	1	3	2
Pc^c^ loci (N)	3	1	4	3	7
LSC	51	47	41	49	48
SSC	6	5	6	6	7
IRa	1	1	1	1	1
IRb	1	1	1	1	1

^a^single-nucleotide SSRs

^b^double-nucleotide SSRs

^c^complex-nucleotide SSRs

The software CodonW was used to calculate and analyze relative synonymous codon usage (RSCU) and bias in the chloroplast genomes. The protein-coding sequences of the *P. mume*, *P. salicina*, and *P. armeniaca* chloroplast genomes consist of 27,632, 27,113, and 28,043 codons, respectively. Among encoded amino acids, leucine is most frequent and tryptophan least frequent. The codon usage bias is related to the genetic information of the ancestral vector, DNA, and proteins involved in biological processes.

### Association analysis between different genes, hotspots, and simple sequence repeats (SSRs)

A comparison of the chloroplast genomes of *Prunus* species suggests a difference in gene arrangement and content, with the *trnl-TAT* gene ranking 10th in the *P. salicina* chloroplast genome and 44th in *P. mume*; in contrast, this gene was not found in *P. armeniaca*. *trnY-ATA* is a unique gene in the *P. armeniaca* chloroplast genome, corresponding to the *ycf1*-*atpB* differential region in the 52–53 k region of the comparison map. Furthermore, *trnH*-*GTG* is at the last position in the *P. armeniaca* chloroplast gene, though it is at the first position in *P. mume* and *P. salicina*. A *trnH*-*GTG*-*matK* hotspot is present in the 0–3 k region. These two differences can be the basis for molecular markers and species identification.

**Fig. 4:**
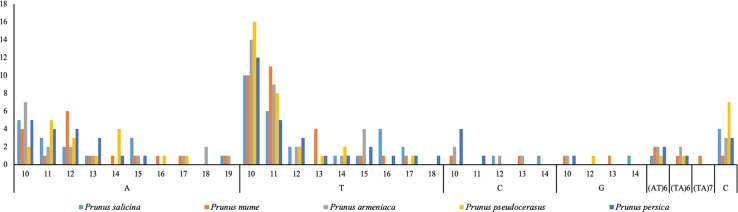
Analysis of simple repetitive sequences in five *Prunus* chloroplast genomes

Using MISA software, we also found 59, 54, 49, 57, and 57 SSRs of at least 10 bp in *P. armeniaca, P. mume*, *P. salicina*, *P. persica*, and *P. pseudocerasus*, respectively (Table 4, Fig. 4). Among these SSRs, most are located in the LSC/SSC region; the IRa and IRb regions have only one SSR. *P. armeniaca* has ten more SSRs than does *P. salicina*. Only single-, double-, and complex-nucleotide SSRs were detected in these *Prunus* species, though no three- or four-nucleotide SSRs were detected. Single-nucleotide repeats in *P. mume*, *P. armeniaca*, and *P. salicina* account for the total number of SSRs, at 90.74%, 89.66%, and 89.80%, respectively. The high variation of SSRs in the chloroplast genome has excellent value for molecular marker studies and plant breeding.

**Fig. 5:**
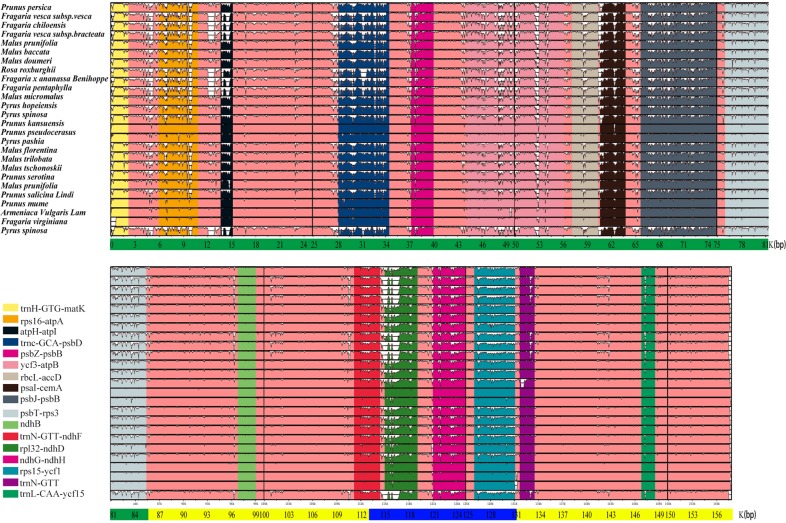
The complete sequence alignment map of 26 chloroplast genomes of Rosaceae. The vertical axis indicates sequence alignment similarity of 50–100%. The green color indicates the chloroplast genome LSC region, the yellow color the chloroplast genome IR region, and the blue color the SSC region. Notes: First grade: *rps16-atpA, atpH-atpI, trnc-GCA-psbD, ycf3-atpB*, and *rpL32-ndhD*. Second grade: *trnH-GTG-matK, PsbZ-PsbB, rbcL-accD, psaI-cemA, psbJ-psbB, psbT-rps3, ndhG-ndhH*, and *rps15-ycf1*. Third grade: *trnL-CAT-ycf15, trnN-GTT, trnR-GTT-ndhF*, and *ndhB*

We divided these regions into three grades based on the degree of difference (Fig. 5). The first grade has significant differences among the 26 Rosaceae chloroplast genomes and includes *rps16*-*atpA*, *atpH*-*atpI*, *trnc*-*GCA*-*psbD*, *ycf3*-*atpB*, and *rpL32*-*ndhD*, which can be used as focus regions for the development of and molecular marker studies in Rosaceae fruit. The second grade is the significant difference in a portion of the 26 Rosaceae chloroplast genomes, including *trnH*-*GTG*-*matK*, *PsbZ*-*PsbB*, *rbcL*-*accD*, *psaI*-*cemA*, *psbJ*-*psbB*, *psbT*-*rps3*, *ndhG*-*ndhH*, and *rps15*-*ycf1*, which can be considered as hotspots for the research and development of molecular markers of Rosaceae. The third grade, comprising the four regions *trnL*-*CAT*-*ycf15*, *trnN*-*GTT*, *trnR*-*GTT*-*ndhF*, and *ndhB*, displays a partial difference among Rosaceae. These 17 regions are generally rich in SSRs, for example, *rps16-atpA* in first-grade hotspots contain six [(A)_10_, (A)_12_, (A)_10_, (T)_10_, (T)_16_, and (T)_10_], six [(A)_17_, (A)_12_, (A)_12_, (A)_10_, (T)_11_, and (T)_11_] and six [(A)_15_, (A)_17_, (A)_10_, (T)_10_, (T)_10_, and (T)_10_] SSRs in *P. mume*, *P. salicina*, and *P. armeniaca*, respectively. The *atpH*-*atpI* hotspots have three [(A)_11_, (T)_12_, and (T)_11_], two [(T)_11_ and (T)_11_], and two [(T)_10_ and (T)_11_] SSRs in *P. mume*, *P. salicina*, and *P. armeniaca*, respectively. The *trnc-GCA-psbD* hotspots have two [(T)_10_ and (T)_10_], one [(A)_12_], and two [(A)_12_ and (T)_10_] SSRs in *P. mume*, *P. salicina*, and *P. armeniaca*, respectively. The *ycf3-atpB* hotspots have four [(AT)_7_aaa(AT)_6_, (T)_10_, (A)_15_, and (T)_10_], five [(T)_11_, (T)_10_, (A)_12_, (TA)_7_, and (T)_10_] and five [(T)_11_, (A)_12_, (TA)_6_, (T)_10_, and (ATA)_5_tact(ATA)_5_] SSRs in *P. mume*, *P. salicina*, and *P. armeniaca*, respectively. The *rpL32-ndhD* hotspots have two [(A)10taaaatatttttcttaattaattatttctgattcaccggttcttatttgttttctgttgaaaggggtcagttaat(A)10 and (A)_15_], one [(A)_14_], and one [(A)_14_] SSRs in the three species. The other two grades are also similar and contain abundant SSR molecular markers, and their distribution is positively related.

In conclusion, the difference in the sequence of the IR region is smaller than that in the LSC and SSC regions. The coding region of the gene is more conserved than is the noncoding region, and the rRNA region is also conserved. Furthermore, the intron region shows the highest mutation rate, followed by the LSC region, the chloroplast genome, the SSC region, and the protein-coding region, with a slight change in the IR region. The distribution of SSRs is positively related to differential hotspots.

### Chloroplast phylogenetic analysis

**Fig. 6:**
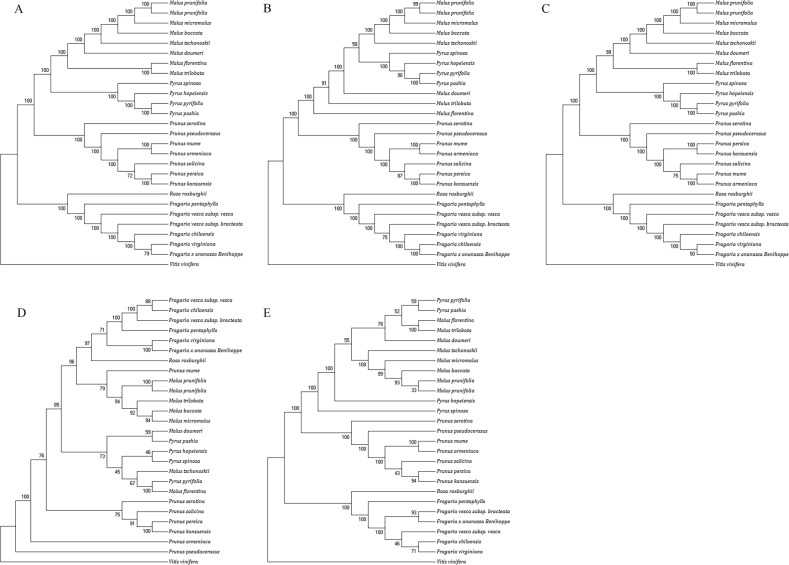
Phylogenetic trees of the *Prunus* species based on the chloroplast genome by MP. **a** Phylogenetic tree constructed using the complete chloroplast genome data. **b** Phylogenetic tree constructed using coding region data. **c** Phylogenetic tree constructed using LSC data. **d** Phylogenetic tree constructed using intron data. **e** Phylogenetic tree constructed using IR data

Phylogenetic relationships of the Rosaceae family and taxonomic statuses were systematically classified through maximal parsimony analysis of three complete chloroplast sequences. In this study, we combined 23 published complete chloroplast genomes and the chloroplast genomes of *P. mume*, *P. salicina*, and *P. armeniaca*. Thus, a total of 26 species were used to reconstruct a phylogenetic tree using MEGA7 software. We utilized different data, including the complete chloroplast genome and CDS, LSC, IR, and intron regions to construct the phylogenetic tree (Fig. 6). The phylogenetic trees constructed with complete chloroplast genome, CDS, and LSC data have the same topology, whereas the trees constructed from IR and intron datasets have low reliability. The phylogenetic trees based on the complete chloroplast genome, CDS, LSC, IR, and intron data have high bootstrap values (this value is generally considered to be a more stable branch than 75). The higher is the branch’s credibility, the more consistent is the guiding value of the evolutionary analysis for the relationship. Furthermore, the phylogenetic trees suggest that *P. mume*, *P. salicina*, and *P. armeniaca* form a single group. Our results showed that the genera *Malus*, *Prunus*, *Fragaria*, and *Rosa* form one branch. *Malus* and *P. salicina* are divided into a taxonomic division, whereas *Fragaria* and *Rosa* belong to a branch that requires further verification. In addition, the phylogenetic trees constructed based on chloroplast genome, CDS, and LSC data showed that *P. mume*, *P. salicina*, and *P. armeniaca* are closer to each other than to other Rosaceae species. The tree branch length of *P. salicina* is long, but those of *P. mume* and *P. armeniaca* are similar; the evolutionary differences between *P. mume*, *P. salicina*, and *P. armeniaca* are pronounced. *P. mume* is closer to *P. armeniaca* than to *P. salicina*.

## Discussion

The chloroplast genome of most angiosperm species contains 74 protein-coding genes, though some have 79 protein-coding genes^16^. Previous studies on Rosaceae fruit trees have revealed that chloroplast gene numbers range from 110 to 130^17^. In this study, sequence analysis revealed 133 genes (110 unique genes), including 94 protein-coding, 31 tRNA, and 8 rRNA genes. The chloroplast genome among *Prunus* species is similar in intron and GC contents, but the GC contents in LSC and SSC regions are significantly lower than that in the IR region. The main reason for this is that all eight rRNA genes with high GC contents are distributed in the IR region. In general, the IR region is the most conserved region of the chloroplast genome^18^. Expansion and contraction in IR, LSC, and SSC regions are common during evolution and are the primary causes of differences in chloroplast genome lengths. A comparative map of the border regions of the chloroplast genome was obtained based on analysis of boundary genes between IR, LSC, and SSC regions. The difference in boundary region size is one of the main reasons for alterations among chloroplast fragments^19,20^.

The chloroplast genomes of the five species of *Prunus* are ~157 kb. *P. salicina* has the largest chloroplast genome at 157,916 bp. In general, there are three main reasons for a change in the size of the chloroplast genome^16^. The first is shrinkage, expansion, or loss of the IR region. Changes in the length of different chloroplast genomes are generally due to changes in the IR region and the boundary between the LSC and SSC regions. Goulding et al.^21^ proposed a hypothesis for the evolution of the chloroplast IR region: there is a small amplification of the boundary gene in the IR zone and recombinant repair of the LSC boundary. Small amplification of the boundary genes in the IR zone is considered to be an important factor in maintaining the stability of the IR zone^22^. The second is loss or increases in genes in the SSC region. The third reason is a decrease in the length of introns or the gene spacer region. For example, the loss of introns in the *ycf1* gene might be the main reason for the smaller chloroplast genome of *P. salicina*. Intron loss was also reported for *Welwitschia mirabilis*, *Hordeum vulgare*, and *Manihot esculenta*^23,24^.

In our study, changes in the IR and boundary between the IR and LSC or SSC of the chloroplast genome of three species were found to be small. The *rps19* gene spans LSC-IR and SSC-IR boundaries, similar to the crossing of LSC-IR and SSC-IR boundaries in the chloroplast genomes of *Ilex pubescens*, *Helwingia himalaica*, and *Panax ginseng*^25^. In some species, the *rps19* gene is located at the border of the LSC/IRa region in the chloroplast genome, as in the genus *Fragaria*;^19^ the boundary in Asteraceae, such as *Millettia pinnata*^26^ and *Lupinus luteus*, is close but does not extend into the IR. In the case of other species, such as *Phaseolus vulgaris*^27^and *Vigna radiata*^28^, the entire gene is present in the IR. The genes ycf1 and ndhF are closest to the SSC-IR border, similar to the case of the *rps19* gene. There are reports of species with genes located on the border, across the border or in the IR region. Comparison between IR regions and the chloroplast genomes of *P. armeniaca*, *P. mume*, and *P. salicina* showed similarity, which was the same as for the boundary gene of LSC/IRa, IRa/SSC, and IRb/LSC regions and the size of genes and fragments in two adjacent regions. The results revealed high similarity among the three species. We also found that the situation at the IRa/LSC boundary was almost the same in *P. armeniaca* and *P. mume*. Therefore, we speculate that the genetic relationship between *P. armeniaca* and *P. mume* is closer than that between *P. mume* and *P. salicina*, and our subsequent phylogenetic analysis validates our inference. We also found that the boundary of the SSC/IRb region displays the greatest difference among *P. mume*, *P. salicina*, and *P. armeniaca* and that is the most variable region.

In a previous report, 17 mutations were found in the cpDNA of 18 *Prunus* accessions via RFLP analysis. Seven mutations, including one length mutation, clustered densely within a region of ~9.1 kb, which includes *psbA* and *atpA*, in the left border of a large single-copy region of *Prunus* cpDNAs. All of these length mutations occurred within the 9.1 kb region between *psbA* and *atpA*. This region might be an intramolecular recombinational hotspot in *Prunus* species^29^.

Differences in the order and content of chloroplast genomes have already been reported for Aquifoliales^30^, Asterales^31^, Bruniales, Apiales, Paracryphiales, and Dipsacales^30^. Additionally, the *trnY-ATA* gene is a unique gene in *P. armeniaca*, whereas *trnI-TAT* has a different order in *P. mume* and *P. salicina*. *trnI-TAT* of *P. salicina* is located at the 10th position of the chloroplast genome, but this gene is at the 44th position in *P. armeniaca*. The *trnH-GTG* gene is situated last in the *P. armeniaca* chloroplast genome, but it is first in *P. mume* and *P. salicina*. Previous reports have been based on differences in *Accd*, *clcp*, and other protein-coding genes, but differences in tRNA genes were discovered first.

The variation in SSR copy numbers in chloroplasts represents an important molecular marker, i.e., cpSSRs, which are widely used in plant population genetics, polymorphism investigations, and evolutionary research. Zhang et al.^32^ used 10 cpSSRs and 16 nuclear SSRs to explain the morphology and differentiation of 42 species of the subgenus *Prunophora*. In the chloroplast genomes of *P*. *mume*, *P. salicina*, and *P. armeniaca*, the number of SSRs was found to be significantly higher than that in other angiosperms, and the content of A/T repeats is far greater than that of G/C repeats, similar to the results of Melotto-Passarin and other studies^33,34^. In addition, SSR loci in these three species differ from those of strawberry, also belonging to Rosaceae, with three and four duplications found, which has also been found among other families such as *Illex*^30^ and Chinese *Juglans*^35^, and mononucleotides, dinucleotides, trinucleotides, tetranucleotides, pentanucleotides, and complex nucleotides have been detected in their chloroplast genomes. The single-nucleotide repeat in cpSSRs that we found can be used to detect polymorphisms at the population level and to compare long-range phylogenetic relationships of different species. Guisinger and Weng^36,37^ found that repetitive sequences might play an important role in chloroplast genome arrangement and sequence variation. In this study, we found a large number of repetitive sequences in the chloroplast genomes of *P. mume*, *P. salicina*, and *P. armeniaca*, especially in the intergenic region, which is consistent with the results of studies on the chloroplast genomes of *Quercus*^38^ and *Holly*^39,40^. These repetitive sequences can be used as important resources for studying differences in chloroplast genes.

Comparison of whole-genome sequences indicated that the different hotspots correlated positively with the distribution of SSRs and that specific genes are also present in hotspots. The different hotspots of plastids have been used to design molecular markers for phylogenetic relationships, such as *rbcL*, *matK*, and *atpB*, which have been widely used in general phylogenetic studies^30^. The diversity of wild cherry, *P. salicina*, was analyzed using chloroplast markers, revealing a certain evolutionary relationship. Indeed, different regions of chloroplast are important for species-level identification of Rosaceae^12,41^. There are many species with similar traits in the family Rosaceae. By comparing chloroplast sequences, we can clearly observe differences in genomes between species at the molecular level and divide species based on chloroplast sequences. The difference in the IR region between the genera *Malus*, *Fragaria*, and *Prunus* is less than that in LSC and SSC regions. Moreover, coding regions are more conserved than are noncoding regions, and rRNA sequences are also conserved. The intron region showed the highest mutation rate, followed by the LSC region, the complete chloroplast genome, the SSC region, and the protein-coding region, with the IR region having the smallest rate. Sequence variations in *P. mume* and *P. armeniaca* are smaller than those in *P. salicina*, similar to the results of the phylogenetic analysis. These hotspots are important molecular marker resources for phylogenetic analysis and identification of Rosaceae plants^42^.

Phylogenetic relationships in Rosaceae have long been problematic because of frequent hybridization, apomixis, presumed rapid radiation, and historical diversification. Plastid phylogenomics offers novel and deep insight into phylogenetic relationships and diversification history among Rosaceae. The development of chloroplast phylogeny and time estimation provides new evidence for future comparative evolutionary studies^43^. Phylogenetic analysis using the chloroplast genome sequence is applied to evaluate evolutionary relationships of species. Our phylogenetic tree was based on complete chloroplast genome, CDS, LSC, IR, and intron data, and the results are consistent with the traditional classification system, indicating that the classification of Rosaceae is generally reasonable. The results of our phylogenetic analysis partially agree with the traditional classification system of Chinese flora, e.g., the genera *Rosa* and *Fragaria*. This suggests that *Rosa* and *Fragaria* are closely related at the molecular level. The fruit, appearance, shape, and other characteristics of *P. mume* are very similar to those of *P. salicina*, though the taste and fragrance are very similar to those of *P. armeniaca*. However, the plants are more resistant to disease. Our phylogenetic tree suggests that *P. armeniaca* is closer to *P. mume* than to *P. salicina*, supporting the grouping of *P. mume* into *P. armeniaca*. With the emergence of more complete chloroplast genome sequences, the chloroplast genome is also expected to help resolve deeper branches of phylogeny. Although there are differences in the phylogenetic tree structure and molecular phylogeny of the Rosaceae family and relationship among various genera, these chloroplast genome sequences will provide genetic information for understanding the evolution of the plastid genome^44^.

## Conclusion

The chloroplast genome size, GC content, and gene number, and order among three *Prunus* species (*P. mume*, *P. salicina*, and *P. armeniaca*) are highly similar to each other. However, there are differences in SSC/IR and LSC/IR boundaries and in the genes *rps19* and *ycf1*, with different expansion lengths in different species. When compared with other genetically related Rosaceae fruit trees, a total of 17 hot spots with significant differences were identified and can be used for the development of phylogenetic markers. The phylogenetic trees were constructed based on chloroplast genome, CDS, LSC, IR, and intron datasets, supporting the close relationship between *P. mume* and *P. armeniaca*. The phylogeny of Rosaceae was comprehensively analyzed. Our results provide a basis for identifying and overcoming phylogenetic problems at the species level.

## Materials and methods

### Plant material

We used *P. mume, P. armeniaca*, and *P. salicina* for genome sequencing. Young, healthy fresh leaves of *P. mume* and *P. salicina* were collected from the National Field Genebank for *P. mume*, Nanjing, Jiangsu Province, China, and fresh leaves of *P. armeniaca* were obtained from Jiangsu Institute of Agricultural Sciences, Nanjing, Jiangsu Province, China. All samples were immediately frozen in liquid nitrogen and stored at −80 °C.

### Chloroplast genome sequencing and assembly

Total genomic DNA was extracted from 100 mg of fresh leaves using a modified CTAB (cetrimonium bromide) method. The DNA concentration (>50 ng µL^−1^) was measured using a NanoDrop spectrophotometer, and fragmentation was achieved using sonication. The fragmented DNA was purified and end-repaired, and sizes were determined by gel electrophoresis. The PCR products were used to produce short-insert (300 bp) libraries using Illumina Nextera XT and, subsequently, a control library quality for sequencing. We sequenced (based on sequencing by synthesis, SBS, technology) the complete chloroplast genome of the three *Prunus* species using the HiSeq™ X10 platform (Illumina, USA) (Genepioneer Biotechnologies Co. Ltd, Nanjing, Jiangsu, China). Raw reads were filtered using the base quality control software NGSQCToolkit v2.3.3 to obtain high-quality reads. We assembled the chloroplast genomes with NOVOPlasty using clean data and annotated them with CpGAVAS^36^. The technology used in this study comprised a combination of de novo sequencing with the *Prunus persica* chloroplast genome as a reference (NCBI accession number NC_014697.1). Finally, Sanger sequencing was used to verify LSC/IR and SSC/IR junctions.

### Genome annotation and sequence alignment

The chloroplast genome sequences were assembled and annotated using the software Dual Organellar Genome Annotator^44^, coupled with manually edited start and stop codons. The three *Prunus* species chloroplast genome maps were drawn in Organellar Genome DRAW^45^, including the two previously sequenced chloroplast genome sequences from *P. persica* and *P. pseudocerasus*, and our three sequences were aligned by MAFFTv7.0.0 to identify the locations of introns and exons, putative start codons, and stop codons; sequences were then manually edited. Base content was analyzed with Bio-Edit software, and the genome annotation included genes, protein-coding genes, tRNA genes, introns, exons, and intergenic spacers; RSCU was analyzed with MEGA 7 software. We used REPuter (http://bibiserv.techfak.uni-bielefeld.de/reputer/) to find and analyze the sizes and locations of forward, reverse, palindromic, and complementary repeats with a minimal length of 20 bp, an identity of 90% and a Hamming distance of 3^46^. SSRs were identified using MISA (http://pgrc.ipk-gatersleben.de/misa/), with thresholds for mononucleotide SSRs of ten repeats and dinucleotide and hexanucleotide SSRs of five repeats. We used CodonW Software to analyze codon usage bias.

Sequence alignment analysis was performed using the online comparison tool mVISTA. We selected 23 Rosaceae plants with similar genetic relationships for blast searches in NCBI.

### Phylogenetic analysis

The chloroplast genomes of 26 Rosaceae species with strong genetic relationships were selected for phylogenetic analysis, and the grape chloroplast genome (NC_007957.1) was selected as the outgroup. The chloroplast genomic sequences of the 23 Rosaceae downloaded from NCBI were manually annotated. We selected IR, LSC, SSC, CDS, and complete chloroplast genome sequences for phylogenetic analysis. Before constructing the phylogenetic tree, we performed multiple sequence alignment using MAFFT software^47^ to obtain aligned chloroplast genomes for phylogenetic analysis. We used complete chloroplast genome sequence, LSC, SSC, IR, and CDS data and maximum parsimony to construct the phylogenetic tree. An MPL analysis was performed using MEGA 7, and a bootstrap test was performed with 1000 repetitions to calculate the maximum parsimony bootstrap value with tree bisection-reconnection branch swapping. Twenty-six species were compared and phylogenetic evaluated.
